# The assembly and comparative analysis of the first complete mitogenome of *Lindera aggregata*

**DOI:** 10.3389/fpls.2024.1439245

**Published:** 2024-09-03

**Authors:** Yujie Shi, Zhen Chen, Jingyong Jiang, Wenwu Wu, Weifu Yu, Shumeng Zhang, Wei Zeng

**Affiliations:** ^1^ Zhejiang Provincial Key Laboratory of Plant Evolutionary Ecology and Conservation, College of Life Sciences, Taizhou University, Taizhou, China; ^2^ Institute of Horticulture, Taizhou Academy of Agricultural Sciences, Linhai, China; ^3^ State Key Laboratory of Subtropical Silviculture, College of Forestry and Biotechnology, Zhejiang Agricultural and Forestry (A&F) University, Hangzhou, China; ^4^ Zhejiang Hongshiliang Group Tiantai Mountain Wu-Yao Co., Ltd., RedRock Group, Taizhou, China

**Keywords:** *Lindera aggregata*, mitochondrial genome, multi-branched conformation, homologous fragments, phylogeny

## Abstract

*Lindera aggregata*, a member belongs to the genus *Lindera* of Lauraceae family. Its roots and leaves have been used as traditional Chinese medicine or functional food for thousands of years. However, its mitochondrial genome has not been explored. Our aim is to sequence and assemble the mitogenome of *L. aggregata* to elucidate the genetic mechanism and evolutionary pathway. The results had shown that the mitogenome was extremely complex and had a unique multi-branched conformation with total size of 912,473 bp. Comprehensive analysis of protein coding genes of 7 related species showed that there were 40 common genes in their mitogenome. Interestingly, positive selection had become an important factor in the evolution of *ccmB*, *ccmFC*, *rps10*, *rps11* and *rps7* genes. Furthermore, our data highlighted the repeated trend of homologous fragment migrations between chloroplast and mitochondrial organelles, and 38 homologous fragments were identified. Phylogenetic analysis identified a tree that was basically consistent with the phylogeny of Laurales species described in the APG IV system. To sum up, this study will be helpful to the study of population genetics and evolution of *Lindera* species.

## 1 Introduction

The genus *Lindera* is a member of the Lauraceae family, comprising approximately 100 species distributed in tropical, subtropical and temperate regions of Asia, as well as temperate regions of North America (Hung-Pin, 1987). There are more than 40 species of this genus in China, accounting for 46% of the whole genus (Tao et al., 2024). The plants of *Lindera* are widely used in traditional medicine and have high economic value, and *Lindera aggregata* (Sims) Kosterm is a representative one of them. It is also known as “Wu-Yao”, is a common folk medicinal plant (Lv et al., 2023).

The leaves of *L. aggregata* are edible and are often used as functional tea or dietary supplements, with health effects such as anti-liver injury and lipid-lowering (Han et al., 2019; Weng et al., 2022). The dried root of *L. aggregata*, as a traditional Chinese herbal medicine, was first recorded in the BenCaoShiYi in the Tang Dynasty, which had the function of regulating qi and relieving pain, warming the kidney and dispelling cold (Cao et al., 2015). It mainly grows in eastern, central, southern and southwestern China, such as Zhejiang, Jiangxi, Anhui and other provinces. Among them, BenCaoGangMu recorded: “The Wu-Yao originated from Tiantai was the best”. Therefore, Zhejiang Tiantai has been a famous genuine producing area of *L. aggregata* since ancient times, which is called “Tiantai Wu-Yao”. The main active components of *L. aggregata* are sesquiterpenes, alkaloids, flavonoids and volatile oils, which have the effects of protecting liver, anti-inflammation, anti-virus, antibacterial, anti-tumor and anti-oxidation (Salleh, 2020). Due to the increasing use of authentic *L. aggregata* and the lack of centralized protection, the reserves of authentic *L. aggregata* are decreasing day by day, so intensive management is extremely urgent. According to the Chinese Pharmacopoeia, “the taproot, which is not spindle-shaped, cannot be used medicinally”. Moreover, the growing year of *L. aggregata* is very long, and the quality of traditional Chinese sold in the marker is often poor, which leads to great difficulties in germplasm selection. Therefore, it is essential to effectively identify authentic medicinal materials in traditional Chinese medicine. In recent years, DNA bar code technology has been widely used in the classification and identification of species, kinship and diversity analysis (Hebert et al., 2003). The primitive herbs used in traditional Chinese medicine are also the object of molecular research (Lu et al., 2016). For example, the *nad7* gene of the mitogenome can be used to identify the Korean ginseng cultivar “Chunpoong” and other ginseng (Wang et al., 2009).

The genus *Lindera* is a large and complex genus with similar transitional characters among different species. In the past, the molecular phylogeny of *Lindera* was mainly focused on the chloroplast genome, which revealed that *Lindera* was a polyphyletic group (Zhao et al., 2018; Shi et al., 2024), and its systematic relationship with related species from *Litsea* and *Laurus* is still unclear. Mitochondrial genome analysis is very important for understanding the evolution and genome structure of various plants (Yang et al., 2023). Recent research on the mitogenome of the genus *Cinnamomum* has further discussed the evolutionary location of the Magnoliids species (Bi et al., 2024b; Han et al., 2024), but information on the mitogenome of other genera from Magnoliids is still lacking. In particular, the mitochondrial genome of *Lindera* plants has not been reported so far. It is an urgent task to analyze the genomic characteristics of *L. aggregata*, compare and analyze the evolution of the mitogenome of *Lindera* and other Laurales plants.

Plant chloroplasts and mitochondria are two important semi-autonomous organelles except for the nucleus, which have their own independent genetic material and genetic system (Gualberto et al., 2014). Mitochondria are important organelles responsible for energy conversion and the main place for aerobic respiration, which play an important role in plant growth and development and stress response (Millar et al., 2005; Wang et al., 2024). Compared with chloroplasts, the size and shape of the mitochondrial genome is very irregular, and its content is equivalent to 1%-2% of the nuclear genome in terms of size. Angiosperms are considered to be the largest group of mitochondrial genomes so far, and the sizes of mitochondrial genomes in different plants vary significantly, ranging from 66 kb to 11.3 Mb (Sloan et al., 2012; Skippington et al., 2015), but the coding genes are relatively conservative (Ogihara et al., 2005). Therefore, the differences in the length of mitogenome from different species mainly depend on the repeat sequences, especially the non-coding regions, including horizontal gene transfer (HGT) (Gandini et al., 2019). Due to the frequent recombination in the mitochondrial genome of higher plants, the structure of the mitogenome is also very different, resulting in the emergence of subring and isomers (Arrieta-Montiel and Mackenzie, 2011). At the same time, the frequently recombined repeat sequences in most plant mitogenome are mainly non-coding DNA, which is closely related to genome recombination (Li et al., 2022). In addition, there are a large number of RNA editing phenomena in higher plant mitochondria, which are one of the necessary steps of plant mitochondrial gene expression (Kubo and Newton, 2008). At present, it has been found that the most types of RNA editing sites are C to U, which mainly occur in the protein coding region, and most editors change the type of amino acids to produce the desired function (Covello and Gray, 1989; Grewe et al., 2011), while the RNA editing sites in some chloroplasts are relatively conservative in evolution (Gray and Covello, 1993; Duan et al., 2018).

In this study, the complete sequence of mitochondrial genome of *L. aggregata* was determined and assembled by the third generation PacBio HiFi sequencing technique. We obtained a multi-branched conformation map of mitogenome and dismantled it into a master circle and a linear fragment. Furthermore, seven mitogenome of Laurales were obtained from NCBI database and compared with the mitogenome of *L. aggregata*, including sequence size, GC content, haplotype network, Ka/Ks, codon usage bias, repeat sequences and RNA editing site prediction. The events of rearrangement in the evolution of mitogenome in 8 Laurales species were compared by using collinear maps. The fragment migrations between chloroplast and mitochondrial genome of *L. aggregata* were also analyzed. Finally, maximum likelihood and Bayesian inference phylogenetic trees were constructed using the conserved PCGs in the mitogenome of 30 species. These results provide valuable information for the mitogenome of *Lindera* and will be of great value to the classification, species identification and molecular breeding of this genus with high value.

## 2 Materials and methods

### 2.1 Sample collection, DNA extraction and sequencing

The sample materials were collected from the planting base of *Lindera aggregata* from Zhejiang Hongshiliang Group Tiantai Mountain Wu-Yao Co., Ltd., Zhejiang, China (120°48.23′ E, 29°12.71′ N). Fresh and tender leaves were collected and quick-frozen with liquid nitrogen. Genome DNA was extracted by improved CTAB method (Doyle and Doyle, 1987). The quality and concentration of total DNA was detected by 1% agarose gel electrophoresis, NanoDrop spectrophotometer and Qubit v4 fluorometer. The qualified high-quality DNA samples (main band > 30 kb) were randomly broken into fragments (15-18 kb) by Covaris ultrasonic crusher; the segmented DNA were enriched and purified by magnetic beads, and the damage repair and end repair were carried out; the stem ring sequencing adapters were connected at both ends of the DNA fragment, and the failed fragments were removed by exonuclease. Finally, the constructed library was used for single molecule real-time (SMRT) sequencing on the PacBio Sequel II platform. The raw reads obtained subreads after removing the adapters and filtering, and the ccs software was used to generate high-precision HiFi reads (91.8 G) with parameter “min-passes = 3, min-rq = 0.99”.

### 2.2 Assembly and annotation of the mitochondrial genome

The mitochondrial genome was assembled by PMAT v1.5.3 software (Bi et al., 2024a), and the initial mitochondrial assembly map was obtained. Bandage v0.8.1 software (Wick et al., 2015) was used to display the mitochondrial genome and manually remove the extended segments of the chloroplast and nuclear genome. Then, the HiFi reads were compared with the mitochondrial genome by Minimap2 v2.24 tool (Li, 2021) to help analyze the repetitive regions in the mitochondrial genome, and finally produce a complete mitogenome of *L. aggregata*. Intelligent Plant Mitochondrial Genome Annotator (IPMGA, http://www.1kmpg.cn/ipmga/) was used to annotate the mitochondrial genome. tRNA and rRNA in mitochondrial genome were annotated by tRNAscan-SE v2.0 (Chan et al., 2021) and BLASTN software (Chen et al., 2015), respectively. The mitochondrial genome was visualized by Plant Mitochondrial Genomes Map (PMGmap) online software (Zhang et al., 2024), and the *cis-* and *trans-*splicing gene maps were drawn. Finally, the mitogenome of *L. aggregata* was uploaded to the GenBank database to obtain the accession number.

### 2.3 Comparative analysis of 8 plants mitogenome from Laurales

We downloaded all the published mitogenome of Laurales (*Cinnamomum camphora*, *C. chago*, *C. chekiangense*, *C. insularimontanum*, *Caryodaphnopsis henryi*, *Machilus pauhoi* and *Hernandia nymphaeifolia*) from the NCBI database and re-annotation of them using the same method mentioned above for statistical and subsequent analysis. The protein coding gens (PCGs) of mitogenome from 7 Laurales plants and *L. aggregata* were extracted by PhyloSuit v1.2.3 software (Zhang et al., 2020). The multi-sequence alignment was performed among 40 common PCGs by MAFFT v7.149b software (Katoh and Standley, 2013), and the sequences were trimmed by trimAl v1.2 software (Capella-Gutiérrez et al., 2009). The trimmed sequences were merged, and the maximum likelihood (ML) phylogenetic tree was constructed by IQ-TREE v2.2.0.3 software (Minh et al., 2020) with default parameters. Furthermore, through the Genepioneer Cloud (http://cloud.genepioneer.com:9929/#/home), the aligned sequences were removed from the sites with gap/missing and the sites with no different bases at the same position in all the sequences, while the remaining bases were haplotype sequences. Then, these haplotype sequences were analyzed using DnaSP v6.12.03 software (Rozas et al., 2003) to generate NEXUS haplotype data files. Haplotype networks of 40 conserved protein coding sequences were constructed via PopART v4.8.4 software (Leigh and Bryant, 2015). The ratio of non-synonymous to synonymous mutations (Ka/Ks) of 40 common PCGs in 8 species mitogenome from Laurales were calculated by KaKs_Calculator v2.0 software (Wang et al., 2010).

### 2.4 Analysis of codon usage bias

In order to analyze the codon usage bias of Laurales species, the relative synonymous codon usage (RSCU) values of PCGs and the number of codon corresponding to each amino acid in 8 species were counted by PhyloSuit v1.2.3 software (Zhang et al., 2020), and visualized by TBtools (Chen et al., 2020). Moreover, in order to determine the GC content and effective number of codon (ENC) of the coding genes, we used the online tool ENC calculation (http://cloud.genepioneer.com:9929/#/tool) to obtain the parameters of 8 species and draw the ENC-plot. The ENC values indicated the extent to which the codon usage of the genome deviates from random selection (Wright, 1990). ENC-plot analysis can explore the relationship between ENC and GC_3_ distribution, which was an effective way to visualize the codon usage bias of genetic data. The standard curve (ENC = 2 + GC_3_ + 29/[GC_3_^2^ + (1 - GC_3_)^2^]) (Romero et al., 2000) indicated that codon usage bias was completely determined by mutation when there was no selection pressure, that was, codon usage bias was completely determined by nucleic acid sequence composition. Each data point in the scatter graph represented a gene. The specific criterion was the distance between the scatters and the standard curve, and the close distance between the scatters and the standard curve indicated that the codon usage bias mainly determined by the base composition and was weakly affected by translation selection; the long distance indicated that the codon ENC value was low and had a strong significant correlation with the level of gene expression, and the codon usage bias strong (Sueoka, 1988).

### 2.5 Analysis of repeat fragments and prediction of RNA editing sites

Simple repetitive sequences (SSRs) were identified by the online tool MISA (https://webblast.ipk-gatersleben.de/misa/) (Beier et al., 2017). The parameters were set as follows: the minimum number of repeats for the mononucleotide repeats, dinucleotide repeats, trinucleotide repeats, tetranucleotide repeats, pentanucleotide repeats and hexanucleotide repeats were set to 10, 5, 4, 3, 3 and 3, respectively. The tandem repeats were identified by Tandem Repeat Finder online tool (https://tandem.bu.edu/trf/home) (Benson, 1999) with default parameters. Dispersed repeats (including forward repeats, reverse repeats, palindromic repeats and complementary repeats) were analyzed by online tool REPuter (https://bibiserv.cebitec.uni-bielefeld.de/reputer) (Kurtz et al., 2001) with the parameters set as follows: minimum repeat length greater than 30 bp, maximum repeat length of 5000 bp and Hamming distance was 3. Moreover, in order to further observe the RNA editing events, the Deepred-mt tool (Edera et al., 2021) was used to predict C-to-U RNA editing sites in 40 PCGs of *L. aggregata*, and the prediction value was set to 0.9.

### 2.6 Identification and collinear analysis of homologous fragments

Based on our previously assembled and annotated chloroplast genome of *L. aggregata* (PP199190) (Shi et al., 2024), the homologous sequences were searched between chloroplast genome and mitochondrial genome using BLAST software with the parameter settings of word size 7 and E value = 1e^−5^. To further study the evolutionary relationship among species, we identified the homologous sequences among *L. aggregata* and other 7 species of Laurales by BLAST program with same parameters as above. The collinear maps within *L. aggregata* (mitochondria and chloroplast) and among different species were visualized by TBtools software (Chen et al., 2020). Furthermore, we used Mauve v2.4.0 software (Darling et al., 2004) to detect the rearrangement of 7 Laurales mitogenome, and the *L. aggregate* mitogenome was used as the reference genome.

### 2.7 Phylogenetic analysis

We downloaded 29 complete mitogenome sequences of 9 different orders (Laurales, Magnoliales, Piperales, Chloranthales, Amborellales, Nymphaeales, Acorales, Poales, and Apiales) from NCBI database. *Amborella trichopoda* and *Nymphaea colorata* were used as outgroups to construct a phylogenetic tree with mitogenome of *L. aggregata*. Firstly, the conserved PCGs were extracted by PhyloSuit software. Then homologous sequence alignment was performed among conserved PCGs in 30 mitogenome by MAFFT software. The Gblocks software was used to trim the aligned sequences. Finally, the phylogenetic trees based on ML and Bayesian inference (BI) were constructed, respectively. The ML tree was built by IQ-TREE software with 5000 ultra-fast bootstraps were set, and the optimal model (GTR+F+I+G4) was obtained by ModelFinder program (Kalyaanamoorthy et al., 2017). The MrBayes v3.2.7a software (Ronquist et al., 2012) was used to construct BI phylogenetic tree, and the best model (GTR+I) was selected by jModelTest tool (Darriba et al., 2012). MCMC was run for 5,000,000 generations, with sampling every 1000 generations. The number of chains and runs was set to 4 and 3, respectively. In the total data, the top 25% of the trees aged and discarded, and the rest were used as a 50% consistent tree; convergence was determined by keeping the average standard deviation of the crossover frequencies < 0.01. ML and BI phylogenetic trees were visualized using Chiplot online tool (Xie et al., 2023). The accession numbers of 30 species were recorded in **Supplementary Table S4**.

## 3 Results

### 3.1 Assembly and annotation of *L. aggregata* mitochondrial genome

**Figure 1:**
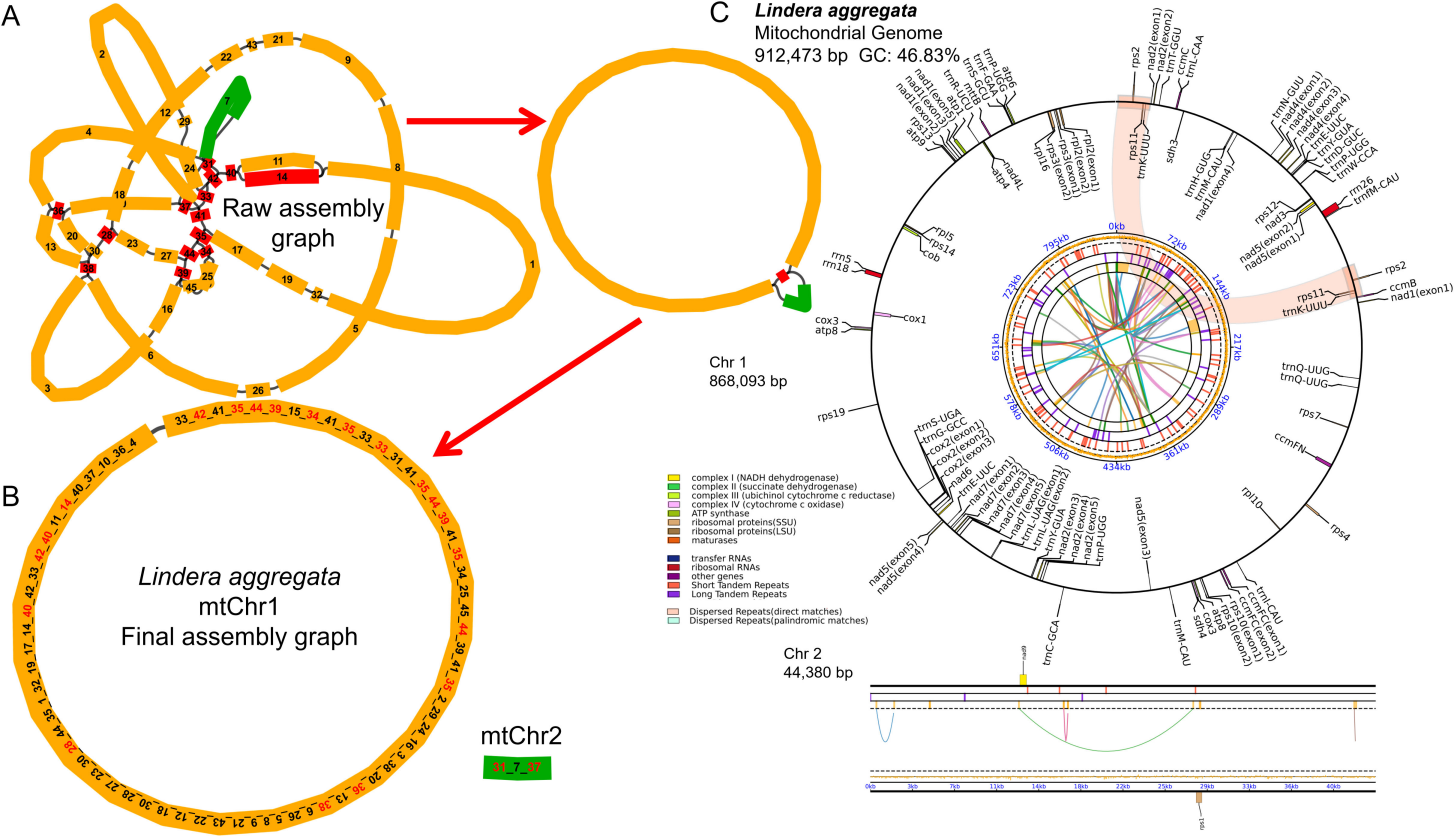
Map of mitochondrial genome assembly and annotation of *L. aggregata.* **(A)** The map of the preliminary assembly of the mitochondrial genome. **(B)** The master circle and linear fragment map of mitochondrial genome. Each contig is marked with different number, and the numbers order of connection is the order of unlocking circles. **(C)** The map of mitochondrial genome annotation. Genes with different functions are described in different colors. The colored parabola in the center circle represents the Dispersed Repeats.

Using the PacBio Sequel II platform, we obtained a total of 5,327,958HiFi reads, with the maximum and average lengths of 59,940 bp and 17,227 bp, respectively. We used HiFi reads to assemble the complete mitochondrial genome of *L. aggregata*. The structure of mitogenome was very complex and had multi-branched conformation (**Figure 1A**), and we analyzed one type of conformation. The total length of the genome sequence was 912,473 bp, the CG content was 46.83%, and it was composed of a longer circular master circle and a shorter linear fragment (**Figure 1B**), with lengths of 868,093 bp (chromosome 1) and 44,380 bp (chromosome 2), respectively. The mitochondrial genome was further annotated, reveled a total of 40 different protein coding genes (PCGs), 28 tRNA genes and 3 rRNA genes (**Figure 1C**; **Supplementary Table S1**). Among the 40 PCGs, 23 were core genes and 17 were non-core genes. The 23 core genes include 5 ATP synthase genes (*atp1*, *atp4*, *atp6*, *atp8*, *atp9*), 4 cytochrome c biosynthesis genes (*ccmB*, *ccmC*, *ccmFC*, *ccmFN*), 1 ubichinol cytochrome c reductase gene (*cob*), 3 cytochrome c oxidase genes (*cox1*, *cox2*, *cox3*), 1 transport membrance protein (*mttB*) and 9 NADH dehydrogenase genes (*nad1*, *nad2*, *nad3*, *nad4*, *nad4L*, *nad5*, *nad6*, *nad7*, and *nad9*). However, it lacked a matures gene (*matR*) as same as the *Cinnamomum* species (Bi et al., 2024b; Han et al., 2024). The non-core genes include 4 large subunits of the ribosomal proteins (*rpl10*, *rpl16*, *rpl2*, *rpl5*), 11 small subunits of the ribosomal proteins (*rps1*, *rps10*, *rps11*, *rps12*, *rps13*, *rps14*, *rps19*, *rps2*, *rps3*, *rps4*, *rps7*) and 2 succinate dehydrogenase genes (*sdh3*, *sdh4*). Furthermore, 8 *cis*-splicing genes (*ccmFC*, *cox2*, *nad4*, *nad7*, *rpl2*, *rps10*, *rps3* and *trnL-UAG*) and 3 *trans*-splicing genes (*nad1/2/5*) were identified. The details of exon in each gene can be found in **Supplementary Figure S1**.

### 3.2 Comparative analysis of mitogenome among *L. aggregata* and other 7 Laurales species

In order to further explore the evolutionary characteristics of the mitochondrial genome, we compared it with seven other species of Laurales (*Cinnamomum camphora*, *C. chago*, *C. chekiangense*, *C. insularimontanum*, *Caryodaphnopsis henryi*, *Machilus pauhoi* and *Hernandia nymphaeifolia*). There were great differences in mitogenome size among 7 Laurales species, ranging from 535,805 bp (*H. nymphaeifolia*) to 1,168,029 bp (*C. henry*), and the GC content ranged from 45.73% (*H. nymphaeifolia*) to 47.12% (*C. chago*) (**Supplementary Figure S2**). In addition, the contents of GC, GC1, GC2 and GC3 of the PCGs were between 44.08% and 44.21%, 49.14% and 49.35%, 44.66% and 45.03%, 38.22% and 38.52%, respectively. For the specific values of each species were found in **Supplementary Table S2**.

It was worth noting that although there were wide differences in the total number of genes in the mitogenome from these eight Laurales plants, the number of PCGs remained relatively consistent (**Supplementary Table S2**). According to the comparative analysis, it can be found that *H. nymphaeifolia* had one more PCGs (*matR*) than other species, and had the largest number of genes (71), while *C. camphora* had the least number of genes (62). The total differences were mainly caused by tRNA, while the number of rRNA all were 3. In order to further analyze these mitogenome, we extracted the coding sequences. 40 shared PCGs were identified in all 8 species and used to construct ML phylogenetic tree (**Supplementary Figure S3**). Interestingly, our results showed that there was a close sister relationship between *L. aggregata* and four species of *Cinnamomum*.

**Figure 2:**
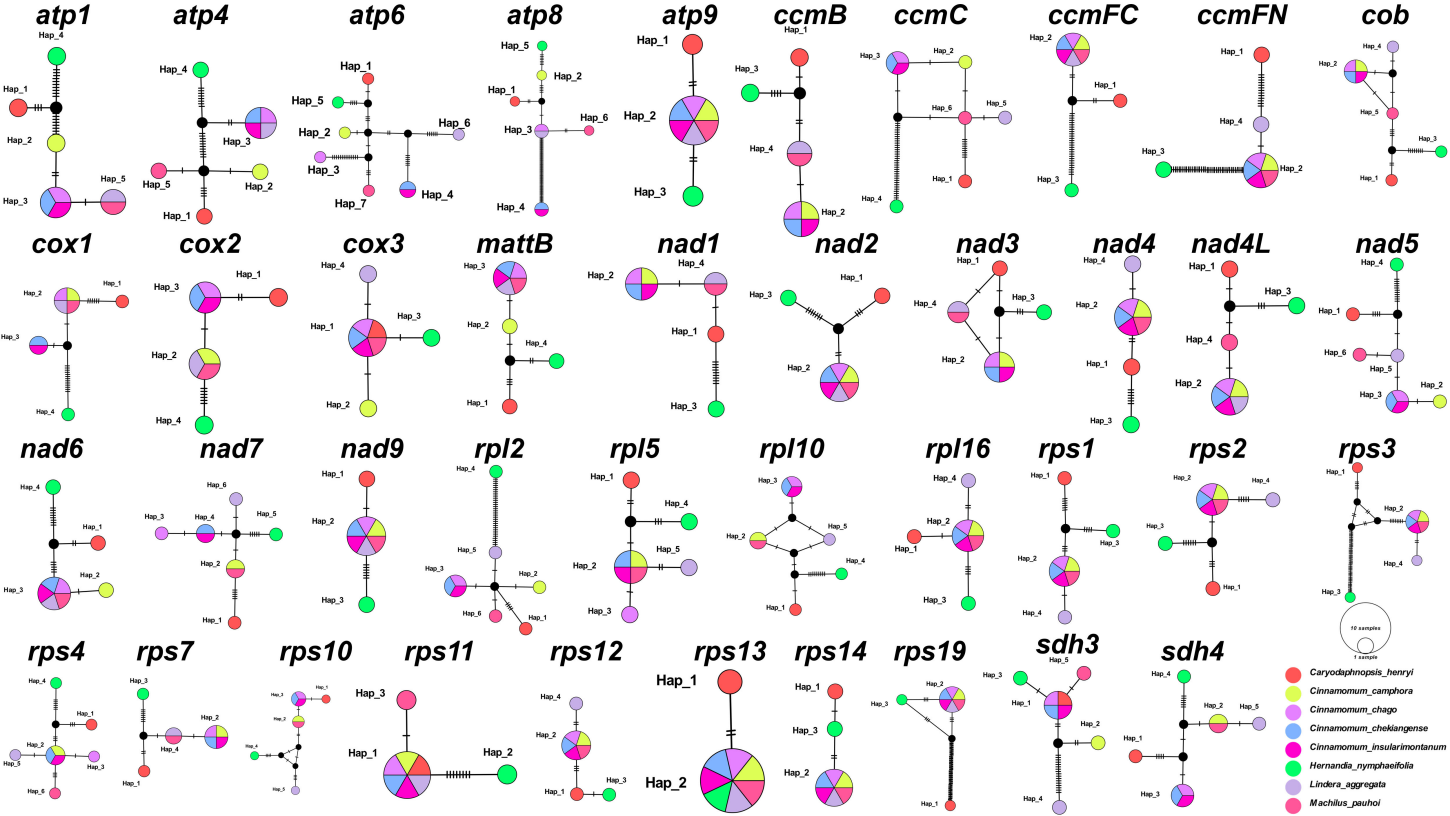
Haplotype networks based on 40 shared PCGs of 8 Laurales species. Different species are given different colors.

We also analyzed 40 common PCGs mutation sites among these 8 Laurales species to construct haplotype networks for more in-depth study of genetic variation (**Figure 2**). It was worth noting that *H. nymphaeifolia* showed the most typical haplotype composition, followed by *C. henryi*, while other species showed more mixed composition. Although *L. aggregata* had 20 unique haplotypes, it also showed mixed haplotypes with *M. pauhoi* and *C. chago*, including 6 haplotypes shared with *M. pauhoi*, 4 haplotypes shared with *C. chago*, and 10 haplotypes shared with 2 of them. Furthermore, *C. insularimontanum* and *C. chekiangense* shared all genes.

**Figure 3:**
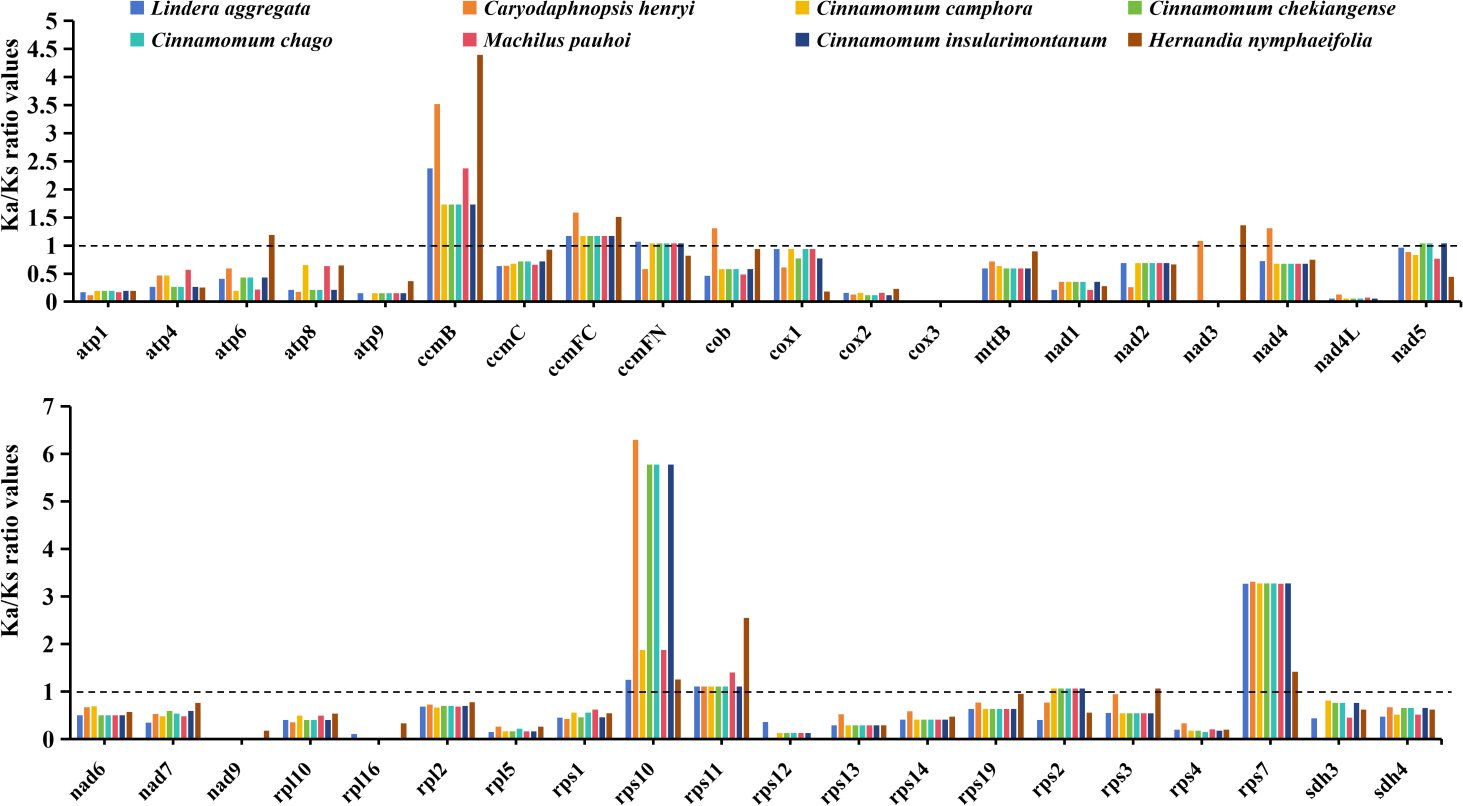
The Ka/Ks ratio values of 40 shared PCGs in the mitochondrial genomes of 8 Laurales plants. Different species are given different colors.

In order to evaluate the effect of environmental stress on the evolution of mitochondrial genome. We calculated the Ka/Ks ratio values of 40 PCGs shared by 8 Laurales species to observe the evolutionary effect of mitogenome under environmental stress (**Figure 3**). The results showed that the Ka/Ks ratio values of most genes were less than 1, indicating that they under purifying selection, while the Ka/Ks ratio values of *ccmB*, *ccmFC*, *rps10*, *rps11* and *rps7* genes were greater than 1 in all species, indicating positive selection. Moreover, a small number of genes were selected in the opposite direction in different species.

### 3.3 Analysis of codon usage bias

**Figure 4:**
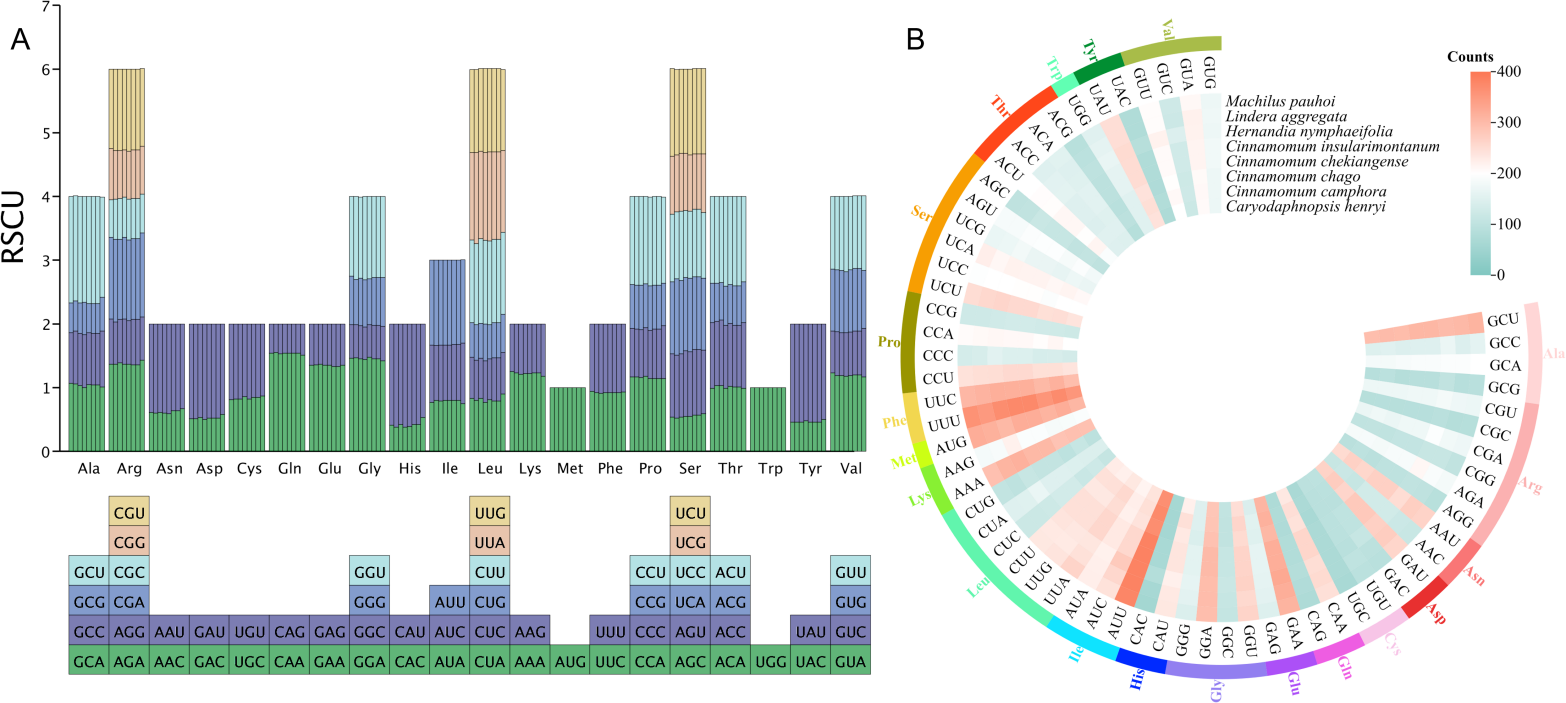
Codon usage bias analysis of 8 mitochondrial genomes in Laurales. **(A)** Analysis of relative synonymous codon usage (RSCU) from 8 species mitogenome. **(B)** The total codon usage of 40 shared PCGs in the mitochondrial genome from 8 Laurales plants.

Codon usage bias analysis of 8 mitochondrial genomes in Laurales showed that 61 codons were detected, encoding 20 amino acids (**Figure 4**). We determined that PCGs had a total of 91,645 codons in 8 mitogenome, distributed between 11,033 and 11,839. Among them, the most frequently used codon was GCU, and the trend was consistent in 8 species. In the 20 kinds of amino acids, serine, leucine and arginine had more codons and had 6 types of codons. Among them, serine had the largest codon number, with 9039 codons (9.86%), followed by leucine with 8780 codons (9.58%), and tryptophan had the least codon number, with only 1241 codons (1.35%) (**Figure 4B**). In addition, the relative synonymous codon usage (RSCU) values of 28 codons in 8 species was greater than 1, indicating that the frequency of these codons was higher, 28 codons were lower than expected (RSCU < 1), while 3 codons (AGU, ACC and ACA) had different usage bias in different species. Interestingly, methionine (AUG) and tryptophan (UGG) did not show codon usage bias (RSCU = 1) (**Figure 4A**). In terms of amino acids, with the exception of AUG (methionine) and UGG (tryptophan), most amino acids showed a bias in their codon usage patterns.

**Figure 5:**
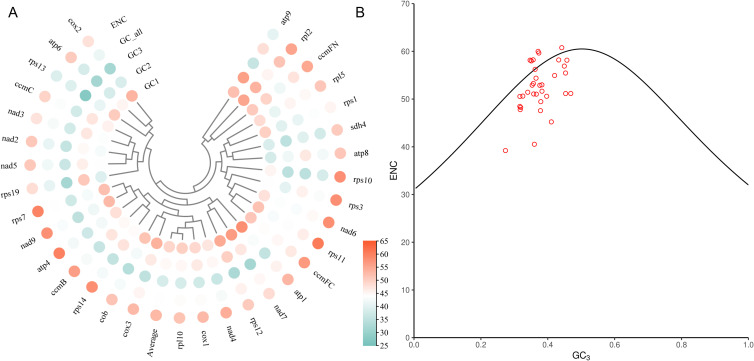
Codon usage bias analysis of PCGs in mitochondrial genome of *L. aggregata*. **(A)** GC content of different sites from 40 PCGs. **(B)** ENC-plot of 40 PCGs. The black curve is the standard curve, the formula is ENC = 2 + GC_3_ + 29/[GC_3_^2^ + (1 - GC_3_)^2^], and each red dot represents a gene.

In order to further evaluate the codon usage bias of each gene in the mitogenome and the factors affecting the usage pattern, we extracted 40 PCGs to calculated the GC content of the first, second and third sites and effective number of codons (ENC) of these genes. The results showed that the GC_1_ values were ranged from 38.12% to 58.24%, the GC_2_ values were ranged from 34.71% to 56%, and the GC_3_ values were ranged from 27.35% to 46.88%. The average contents of GC in different locations (GC_1_, GC_2_ and GC_3_) were all less than 50%, indicating that there was a bias for A/T base and A/T ending codon in the mitogenome of *L. aggregata*. The values of ENC were between 39.21% and 60.77%, with an average of 52.98%, indicating that there was a weak codon usage bias in the mitogenome of *L. aggregata* (**Figure 5A**). Furthermore, through ENC-plot analysis, the results showed that most PCGs were far from the standard curve, and only a small number of genes were distributed beside the curve (**Figure 5B**), indicating that there was a strong significant correlation between most genes and the level of gene expression, and the codon had preference, which was consistent with the above results (**Figure 4**).

### 3.4 Analysis of repeat fragments and prediction of RNA editing sites

**Figure 6:**
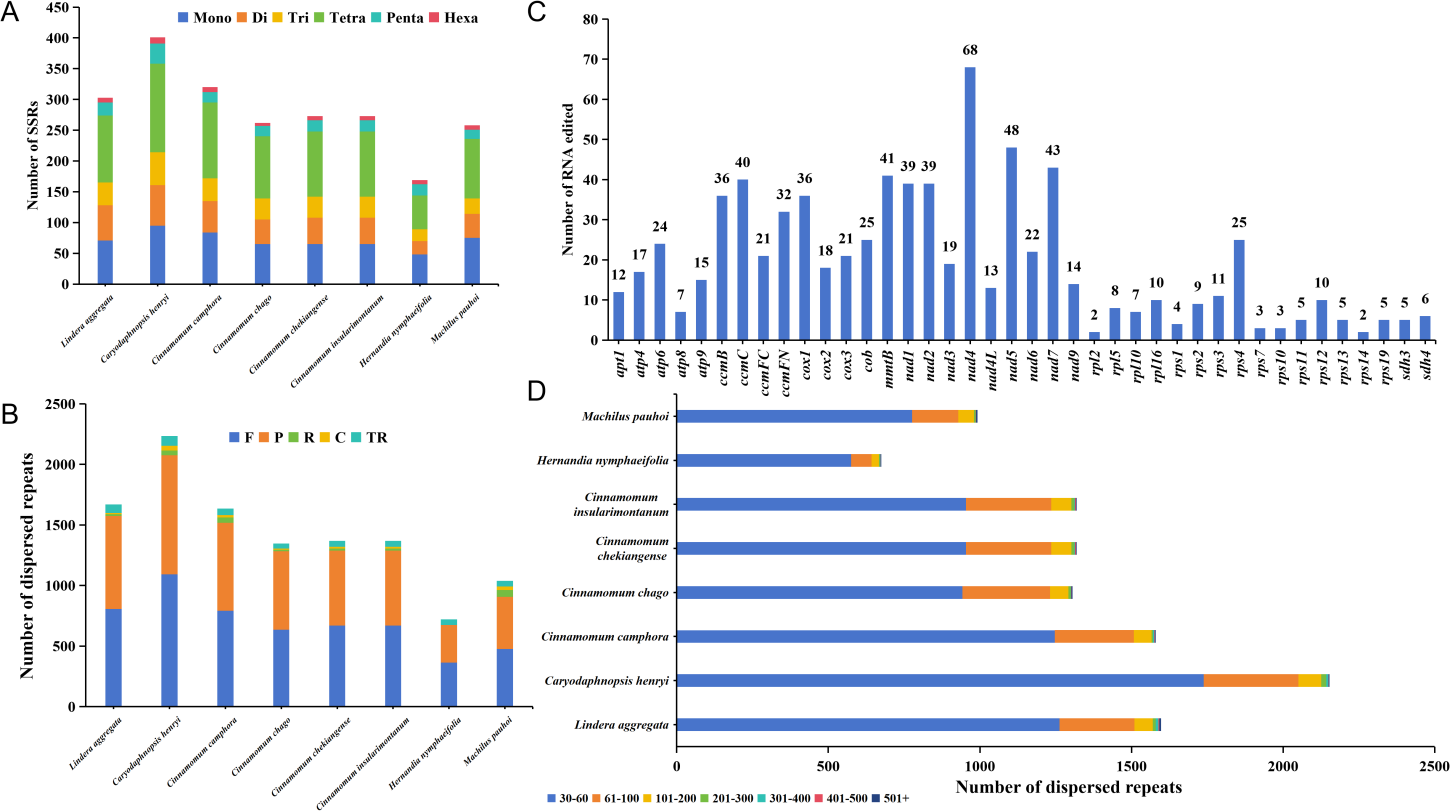
Repeat fragment analysis and prediction of RNA editing sites of mitochondrial genome. **(A)** Detection of mitogenome SSRs in 8 species of Laurales. Mono, Di, Tri, Tetra, Penta, and Hexa represent mononucleotide repeats, dinucleotide repeats, trinucleotide repeats, tetranucleotide repeats, pentanucleotide repeats and hexanucleotide repeats, respectively. **(B)** Prediction of dispersed and tandem repeats of mitogenome in 8 species of Laurales. F, R, P, C, TR represent forward repeats, reverse repeats, palindromic repeats, complementary repeats, and tandem repeats. **(C)** Prediction of RNA editing sites in the mitogenome of *L. aggregata*. **(D)** The size distribution of dispersed repeats in 8 Laurales mitogenome.

Simple sequence repeats (SSRs) were widely distributed in the mitochondrial genome. There were 169-401 SSRs detected across each of mitogenome from 8 Laurales species, for a total of 2259 SSRs. Although there were great differences in the number of SSRs among the 8 Laurales species, they all had 6 constituent types, and the occupation trend of each type was relatively consistent in each species (**Figure 6A**). Among them, the number of tetranucleotide repeats was the largest (55-144 loci), accounting for 32.54%-38.83% of the total SSRs. The number of hexanucleotide repeats was the least (5-10 loci), accounting for 1.91%-4.14% of the total SSRs. Furthermore, we also detected tandem and dispersed repeats in the mitogenome of 8 species. Among them, the distribution of tandem repeats was between 40 and 81, and there was a great difference in quantity. The number of dispersed repeats ranged from 675 to 2153, in which except *H. nymphaeifolia* only had forward (F) and palindromic (P) repeats, the other species contained four types repeats, and the number of F and P repeats were far more than reverse (R) and complementary (C) repeats (**Figure 6B**). The distribution trend of dispersed repeat sequence lengths among the 8 species was consistent, mainly between 30 and 60 bp, accounting for 72.13%-85.19% of the total (**Figure 6D**). Moreover, *C. henryi* and *H. nymphaeifolia* had the most and fewest repeat fragments respectively, which might be an important reason for the size of the mitochondrial genome.

In this study, we also predicted all possible C-to-U RNA editing sites in the *L. aggregata* mitogenome to reveal more deeply the gene expression in the mitochondrial genome. A total of 770 RNA editing sites were found, mainly involving the transition from nucleotide C to U (**Figure 6C**). It is worth noting that the *nad4* gene showed the highest RNA editing frequency, predicting 68 editing sites, followed by the *nad5* gene with 48 editing sites. However, most ribosomal proteins had a small number of editing sites, which was consistent with *C. chekiangense* and *C. camphora* (Bi et al., 2024b; Han et al., 2024). In addition, we observed that RNA editing events mainly occurred at the first and second base positions, and the second base positions changed more frequently. These editing events lead to changes in amino acids, such as Proline (P) to Serine (S) or Leucine (L), Serine (S) to Phenylalanine (F) or Leucine (L), Arginine (R) to Cysteine (C), Tryptophan (Y) or Termination codon (*), Leucine (L) to Alanine (A), Threonine (T) to Isoleucine (I) or Methionine (M), Histidine (H) to Tyrosine (Y), Glutamine (Q) to Termination codon (*), Alanine (A) to Valine (V). Interestingly, most of these amino acid changes were related to the transformation of hydrophobic amino acids, which helped to improve the stability of proteins.

### 3.5 DNA fragment transfer analysis between mitochondrial and chloroplast genomes

**Figure 7:**
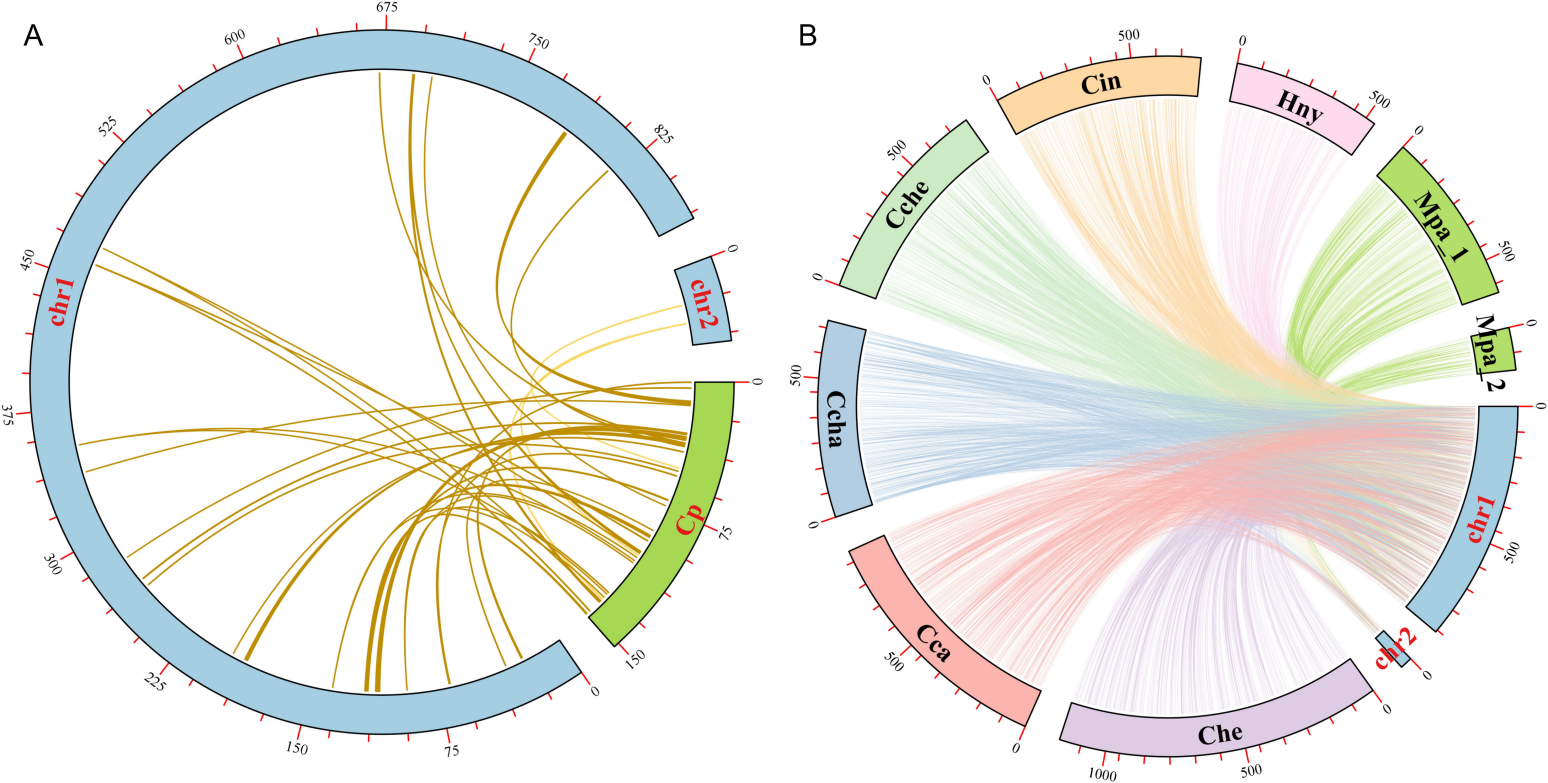
Collinearity analysis among different Laurales species. **(A)** Sequences transfer analysis of chloroplast genome (cpDNA) and mitochondrial genome in *L. aggregata*. The blue and green arcs represent mitogenome and cpDNA, respectively. Homologous fragments are represented by a yellow line between blue and green arcs. **(B)** Collinearity analysis between the mitogenome of *L. aggregata* and other 7 mitogenome of Laurales species. The arcs of different colors represent different mitochondrial genomes and are abbreviated to represent different species. *Lindera aggregata* (chr1 and chr2), *Cinnamomum camphora* (Cca), *Cinnamomum chago* (Ccha), *Cinnamomum chekiangense* (Cche), *Cinnamomum insularimontanum* (Cin), *Caryodaphnopsis henryi* (Che), *Machilus pauhoi* (Mpa_1 and Mpa_2) and *Hernandia nymphaeifolia* (Hny). The homologous fragments between 7 species and *L. aggregata* are represented by their corresponding colors, respectively.

In the evolution of higher plants, the transfer of genetic material between mitochondria and chloroplasts were a common phenomenon. However, it was worth noting that these sequence fragments originating from chloroplast organelles showed relatively low conservatism. Therefore, we searched the homologous fragments between the two organelles in order to explore the migration of sequences from chloroplasts to mitochondrial organelles (**Figure 7A**). Through sequence similarity analysis, a total of 38 homologous fragments were identified between chloroplast and mitochondrial genomes, with sequences length ranged from 32 bp to 1721 bp, with a total of 12,022 bp, accounting for 1.32% of the total mitogenome (**Supplementary Table S3**). The number of mismatches in these fragments varied from 0 to 119, and the number of gaps varied from 0 to 33. These fragments represented the migrations from chloroplasts to mitochondrial organelles, called MtPts. Of the 38 migration segments, MtPt-1 was the longest. We annotated these fragments and identified 15 complete genes, including 2 PCGs (*petN* (2)) and 13 tRNA genes (*trnA-UGC*, *trnD-GUC*, *trnE-UUC*, *trnH-GUG*, *trnI-CAU*, *trnM-CAU*, *trnN-GUU* (2), *trnP-UGG*, *trnR-UCU*, *trnT-GGU*, *trnW-CCA*, *trnY-GUA*). It was worth noting that the *petN* gene in the cpDNA contributed subunits of cytochrome b/f complex, while 13 tRNA genes might have been lost or experienced pseudogene changes in the cpDNA.

### 3.6 Collinearity analysis and structural rearrangement

In order to further study the homologous sequences of the mitogenome in 8 Laurales species, we identified the collinearity between *L. aggregata* and other Laurales species (**Figure 7B** and **Supplementary Figure S4**). The results showed that *L. aggregata* had a large number of collinear fragments with other 7 species in Laurales. Notably, the highest collinearity was detected between *L. aggregate* and *C. camphora*, which contained 1051 collinearity fragments totaling 598,706 bp, accounting for 65.61% of *L. aggregate* mitogenome. However, *H. nymphaeifolia* exhibited the weakest collinearity, with only 378 collinearity fragments spanning 234,099 bp, covering 25.65% of *L. aggregate* mitogenome. It was worth noting that although rearrangement events occurred frequently in intergenic regions, the protein-coding regions of the plant mitochondrial genome were still highly conserved. In the mitochondrial genomes of 8 species, most protein-coding regions overlap with colinear fragments. The most extensive collinearity was detected between *L. aggregate* and *C. camphora*, containing 99.15% of the protein-coding sequences from *C. camphora* mitogenome, while it was relatively lower with *H. nymphaeifolia* (95.93%). This showed that although the PCGs of these 8 Laurales mitogenome had high similarity in sequences, the overall mitogenome structure was unstable.

### 3.7 Phylogenetic analysis of different orders mitochondrial genomes

**Figure 8:**
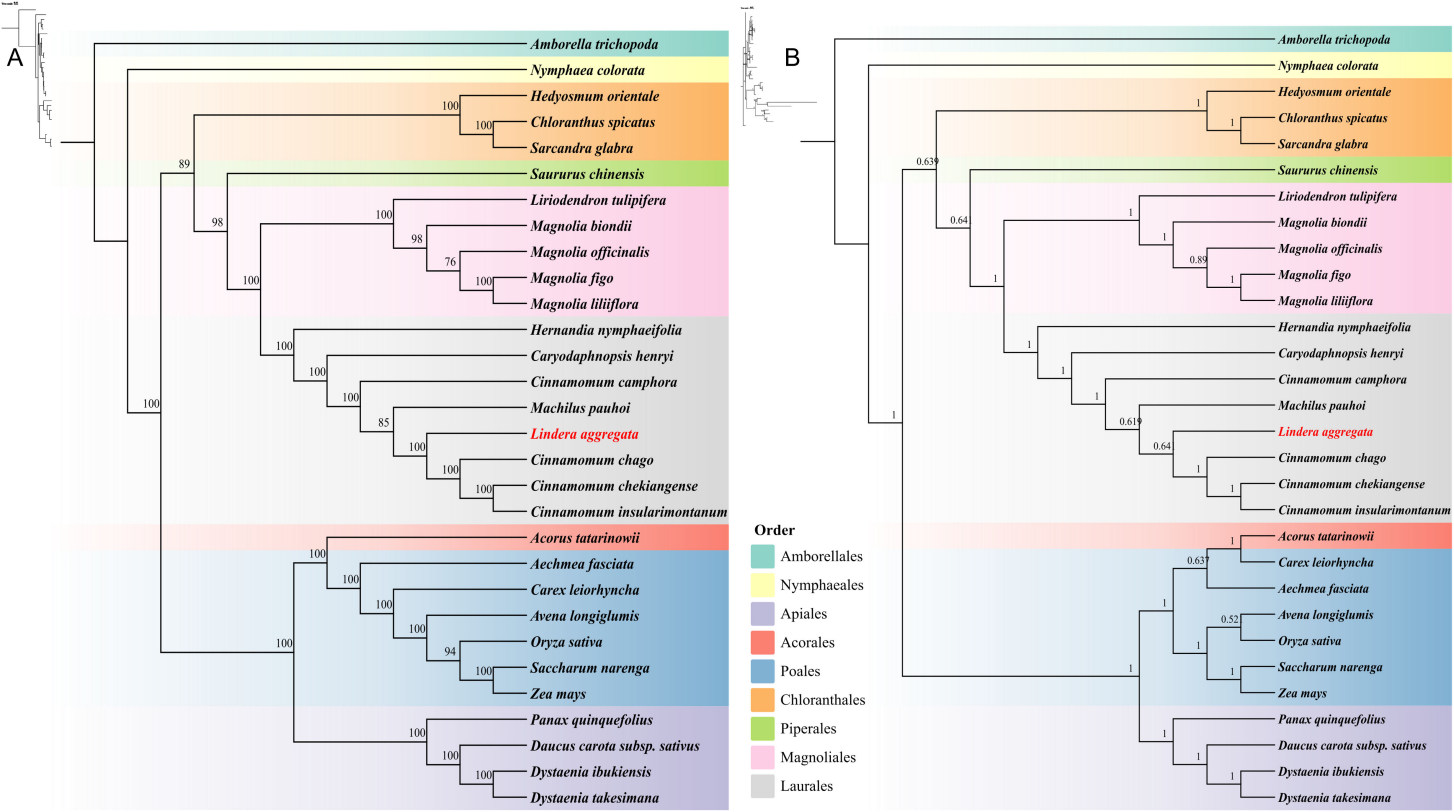
Phylogenetic trees of 30 plant mitochondrial genomes from 9 different Orders. **(A)** The maximum likelihood phylogenetic tree based on 24 common PCGs of 30 species. **(B)** The Bayesian inference phylogenetic tree based on 24 common PCGs of 30 species. The support rate values are shown on the branches on the trees.

We constructed phylogenetic trees based on 24 conserved PCGs (*atp1/4/6/8/9*, *ccmB/C/FN*, *cox1/2/3*, *cob*, *mttB*, *nad1/2/3/4/4L/5/6/7/9*, *rps12* and *sdh4*) of 30 species from 9 different orders (Amborellales, Nymphaeales, Apiales, Acorales, Poales, Chloranthales, Piperales, Magnoliales, and Laurales) in angiosperms. The topologies of maximum likelihood (ML) and Bayesian inference (BI) phylogenetic trees based on mitogenome were basically the same, which could closely reflect the taxonomic relationship between these species (**Figures 8A, B**). It was worth noting that the mitogenome of 8 Laurales species formed a separate branch and together with Magnoliales and Piperales to form Magnoliids. The overall structure of the phylogenetic trees based on mitogenome was completely consistent with the latest classification based on the Angiosperm Phylogeny Group (APG IV). Our results showed that *L. aggregata* belonged to Laurales and represented a relatively new differentiation. In addition, *L. aggregata* was closely related to *M. pauhoi* and *C. chago*, which was consistent with the results of the haplotype network mentioned above.

## 4 Discussion

Most mitochondrial genomes in higher plants have complex circular or linear structures due to frequent recombination mediated by repeat sequences, which can promote the formation of large/small subrings and isomers in plants (Fujii et al., 2010; Smith and Keeling, 2015). Recent studies have revealed great differences in the structure of mitogenome among different species. For example, the mitogenome of *Angelica biserrata* consists of six independent circular chromosomes, while the mitogenome of *Mentha spicata* consists of one linear chromosome and two circular chromosomes, each of which varies in size (Jiang et al., 2023; Wang et al., 2024). In this study, we sequenced, assembled and reported the complete mitochondrial genome of the important Chinese herbal medicine Wu-Yao for the first time. Its mitogenome consists of a master circle (868,093 bp) and a linear fragment (44,380 bp) with a total length of 912,473 bp. A total of 64 unique functional genes were identified in the mitogenome, including 40 PCGs, 21 tRNA genes and 3 rRNA genes. It was worth noting that the GC content of PCGs played an important role in determining amino acid composition in the evolution of terrestrial plants, and the GC content of the mitogenome in most higher plants was between 43% and 45% (Kubo and Mikami, 2007). The GC content of the PCGs in the mitogenome of *L. aggregata* was 44.10%, which was basically consistent with the GC content of the mitogenome from 7 related plants (44.08%-44.21%). The Ka/Ks analysis is an important evaluation method to understand species adaptability, selection factors, evaluate adaptive potential and study heredity and evolution. It is also an important evaluation index to understand how plant genes respond to environmental stress in the process of evolution (Xie et al., 2019). In our comparison, we found that most of the 40 common PCGs in 8 Laurales mitogenome were under negative selection, only a few genes (*ccmB*, *ccmFC*, *rps10*, *rps11* and *rps7*) showed signs of positive selection in all species, while some genes (*atp6*, *ccmFN*, *cob*, *nad3*, *nad4*, *nad5*, *rps2* and *rps3*) were affected by positive selection in individual species. The *atp6*, *ccmB*, *ccmFC*, *ccmFN*, *cob*, *nad3*, *nad4* and *nad5* genes may play an important role in redox reaction and other adaptation processes. The relatively high Ka/Ks ratio values of *rps10* and *ccmB* genes indicated that they played an important role in species evolution and may be closely related to environmental stress.

Codons play an important role in identifying and transmitting genetic information of organisms and connecting proteins and DNA, and also play an important role in genetic and variation of organisms (Andargie and Congyi, 2022; Xiao et al., 2024). Due to the influence of gene mutation and natural selection, some synonymous codons are often used frequently in the process of protein translation, which leads to codon usage preference. This preference for specific synonymous codons plays a vital role in shaping the genetic characteristics of these organisms (Hershberg and Petrov, 2008; Parvathy et al., 2022). In this study, we used 40 PCGs to evaluate the GC content and RSCU of mitogenome in 8 species from Laurales. The results showed that all the PCGs of these species had a preference for A/T base and A/T ending codon. This result is consistent with the previous studies on the mtNDA of *Angelica* and *Mangifera* species (Niu et al., 2022; Wang et al., 2024), indicating that there are some similarities in the codon usage bias of the mitogenome in different species. Moreover, in our previous study, we also found that the third position of the chloroplast genome had a similar trend to the mitogenome in codon usage pattern. This further highlights the common codon usage bias between mitogenome and cpDNA, and emphasizes the high similarity in codon usage preference between the two genetic components (Zhang et al., 2018; Wang et al., 2020; Tang et al., 2022). ENC-plot analysis and RSCU results showed that there was a preference for codon usage in *L. aggregata* mitogenome, and there was a strong correlation between codon usage and gene expression level. It is more affected by natural selection, which is consistent with previous findings in Chinese angelica and soybean plants (Tang et al., 2020; Wang et al., 2024).

The collinear analysis of homologous fragments among 8 species of Laurales showed that the mitogenome of *L. aggregata* and its related species had experienced a large number of rearrangement events. The discovery of these homologous collinear fragments has contributed to the evolution of the genome and the regulation of gene expression. In addition, the proportion and arrangement of repetitive fragments in plant mitogenome play an important role in promoting species evolution, and are also the dominant factors in the size of mitochondrial genome (Pfeifer et al., 2013; Li et al., 2022). A total of 303 SSRs,71 tandem repeats and 1598 dispersed repeats were detected in the mitogenome of *L. aggregata*. These repetitive fragments constitute the intricate branch structure of the mitochondrial genome of *L. aggregata*. It had been found that the gene transfer of homologous fragments in the mitochondria and plastid organelles of a single species and the horizontal transfer between the same organelles of different species highlighted the intercorrelation and flowing genetic structure of these organelles, which was a common phenomenon in higher plants (Gualberto et al., 2014). For example, there is a similar trend of pseudogenes loss or alteration in the chloroplast genome of the *Cistanche* genus, affecting five genes related to photosynthesis and energy production (Miao et al., 2022). In our study, 38 fragments migrated from chloroplasts to mitochondria were successfully identified, and their lengths ranged from 32 bp to 1721 bp, accounting for 1.32% of the total mitochondrial genome length. Further annotation of these homologous fragments revealed 15 complete genes, including 2 PCGs (*petN* (2)) and 13 tRNA genes (*trnA-UGC*, *trnD-GUC*, *trnE-UUC*, *trnH-GUG*, *trnI-CAU*, *trnM-CAU*, *trnN-GUU* (2), *trnP-UGG*, *trnR-UCU*, *trnT-GGU*, *trnW-CCA*, *trnY-GUA*). It was worth noting that the *petN* gene in the cpDNA taken part in the formation of subunits within cytochrome b/f complex, while 13 tRNA genes might have been lost or experienced pseudogene changes in the cpDNA.

RNA editing is a common post-transcriptional phenomenon, which widely exists in the organelles of angiosperms, including insertion, deletion and substitution, leading to changes in genetic information (Sun et al., 2016). Studies have shown that RNA editing is mainly composed of base substitutions mediated by deaminase, in which cytosine substitutes for uracil (U-to-C) have the highest frequency, and a few of them are uracil substituted cytosine (C-to-U) and hypoxanthine substituted adenine (A-to-I) (Zhu et al., 2022). The identification of RNA editing is very important for the study of mitogenome in *L. aggregata*. Because RNA editing can change amino acids, it often increases the overall conservatism of amino acids and changes their physical and chemical properties, thus affecting the function of proteins. Previous studies have shown that the presence of hydrophilic amino acids can promote protein folding, which is related to the decrease of the overall stability of protein structure (Gray, 2009; Bi et al., 2019). A total of 770 RNA editing sites were identified in the mitogenome of *L. aggregata*, of which the number of editing event of *nad4* gene was the most (68 sites), while there were generally few RNA editing sites for genes related to ribosomal proteins. Furthermore, most of the amino acids in the mitogenome were transformed from hydrophilic amino acids to hydrophobic amino acids, resulting in the increase of protein hydrophobicity and overall structural stability. In order to further understand the close relationship between Laurales plants and other angiosperms, we used 24 shared PCGs of 30 species from 9 orders to construct phylogenetic trees. The results showed that it was consistent with the phylogenetic tree of APG IV and accurately showed the taxonomic relationship of angiosperms. It was worth noting that *matR* gene was missing from the mitogenome of Lauraceae species, while *matR* gene was found in its closest relatives, Hernandiaceae species. This shows that *matR* gene is lost after the differentiation of Lauraceae and Hernandiaceae, and can be used as an important marker to distinguish Lauraceae species from other species, and it may be great significance to the adaptive evolution of Lauraceae species.

## 5 Conclusion

In this study, we completed the assembly and annotation of the mitogenome of *L. aggregata*. The mitogenome showed a branch structure of 912,473 bp in length. Comparative analysis showed that the mitogenome of *L. aggregata* contained abundant repeated sequences and HGT events, which might be one of the important reasons for the difference in the size of the mitogenome of *L. aggregata*. Furthermore, the mitogenome of Laurales species had experienced frequent rearrangement events, and the non-coding regions showed poor collinearity, but the structure of the coding regions was very conservative. Phylogenetic analysis based on 30 mitochondrial PCGs showed that Magnoliids was sister group of the monocots and eudicots taxa, and the evolutionary position of *Lindera* species in Magnoliids. This study will lay a foundation for the application of population genetics and evolution of *Lindera* and other species from Magnoliids.

## Data Availability

The accession numbers of mitochondrial genome of *L. aggregata* in Gene Bank are PP848112 and PP848113. The raw HiFi sequencing data have been deposited in NCBI with accession number: SRR30136615.
