# Supplementary material for: The assembly and comparative analysis of the first complete mitogenome of Lindera aggregata
Source: Front Plant Sci. 2024 Sep 3;15:1439245. doi: 10.3389/fpls.2024.1439245 (PMC11405213; doi:10.3389/fpls.2024.1439245)
Supplement: Supplementary file 1 [file Table1.docx]

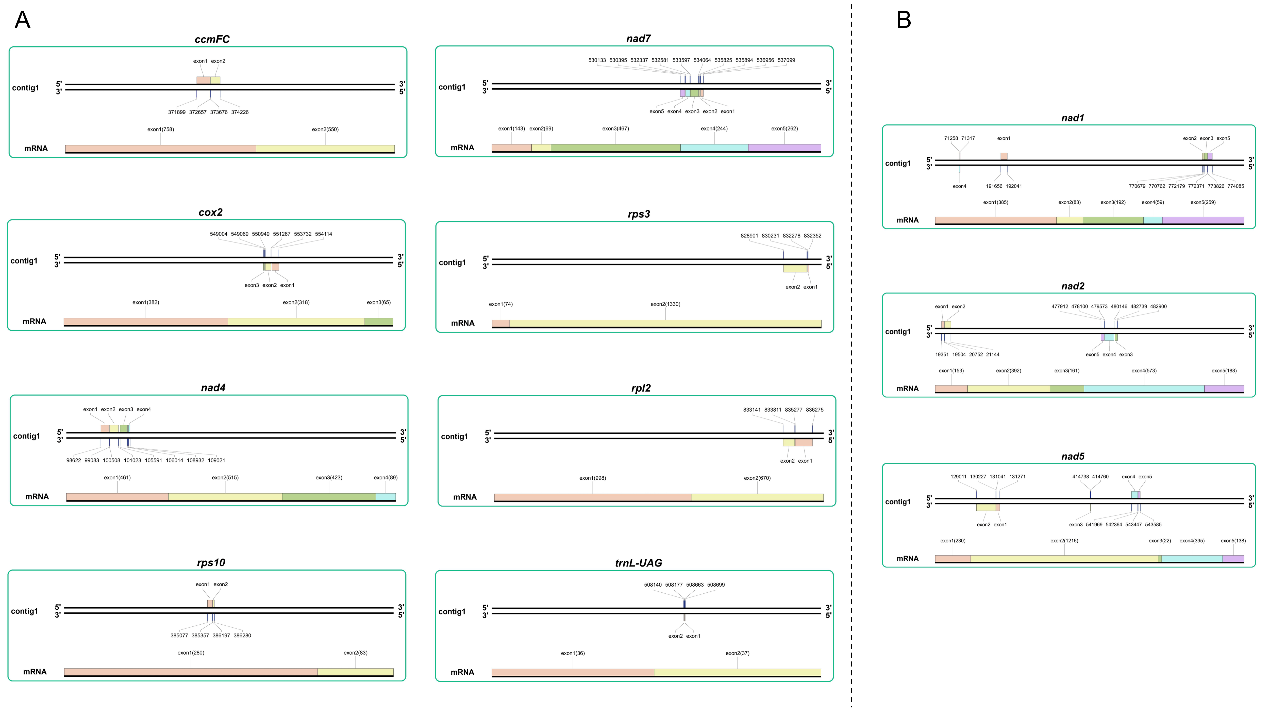


Figure S1. Identification of cis-splicing genes (A) and trans-splicing genes (B) in the mitochondrial genome of *L. aggregata*

*
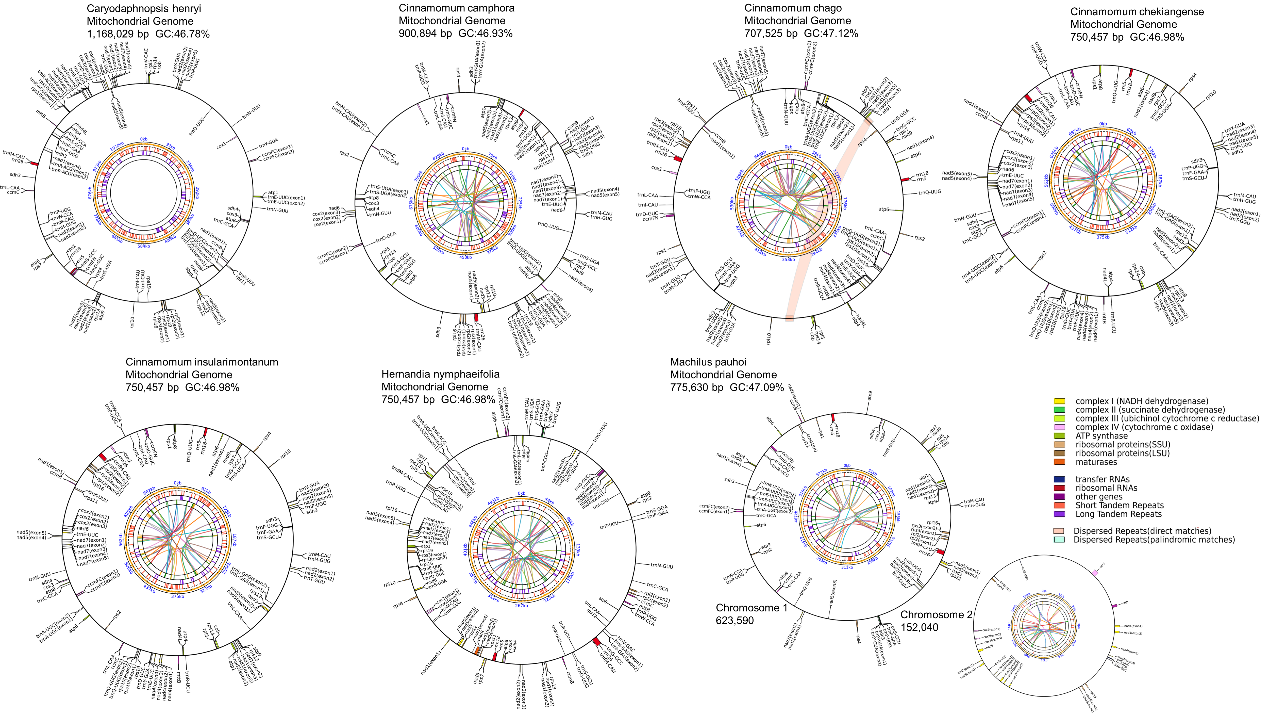
*

Figure S2. The map of mitochondrial genomes from 7 species (*Cinnamomum camphora, C. chago, C. chekiangense, C. insularimontanum, Caryodaphnopsis henryi, Machilus pauhoi* and *Hernandia nymphaeifolia*) in Laurales. Genes with different functions are described in different colors. The colored parabola in the center circle represents the Dispersed Repeats.

*
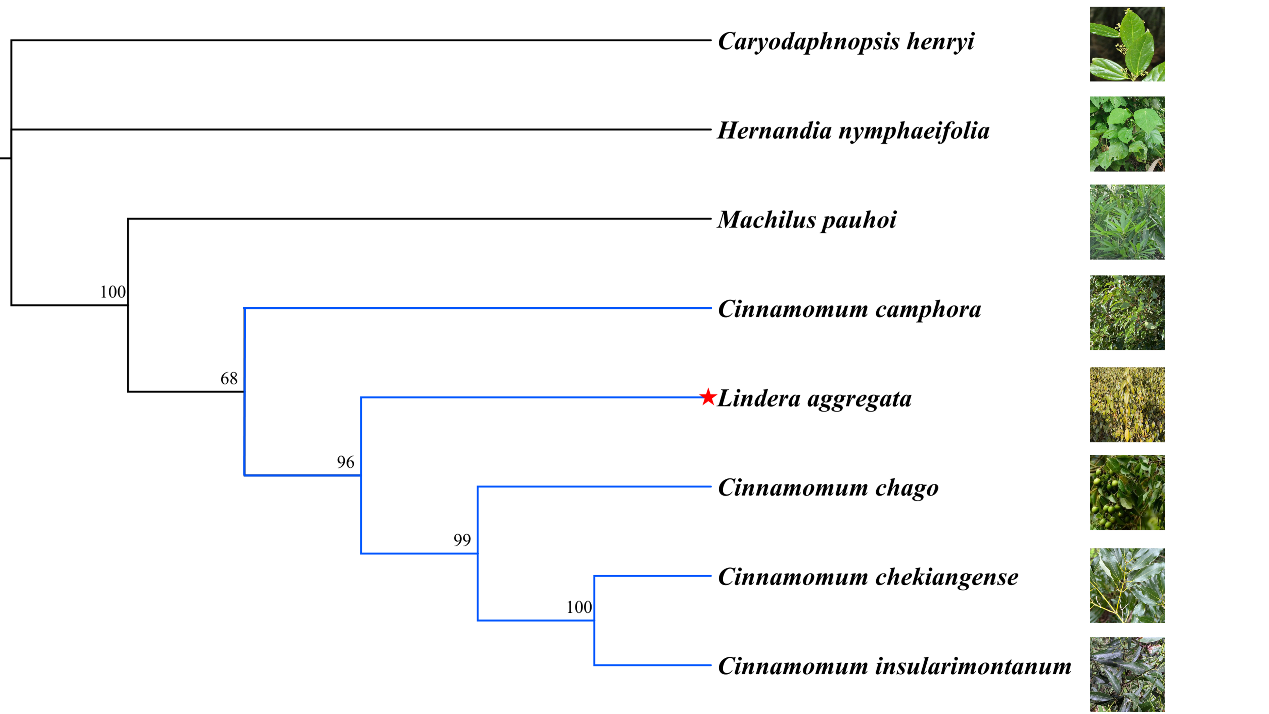
*

Figure S3. The ML phylogenetic tree was constructed based on the mitochondrial genomes of *L. aggregata* and 7 other species from Laurales. The *L. aggregata* was highlighted with a red pentagram, while the blue-branched species were most closely related to it. The support rate values were shown on the branches of the tree.


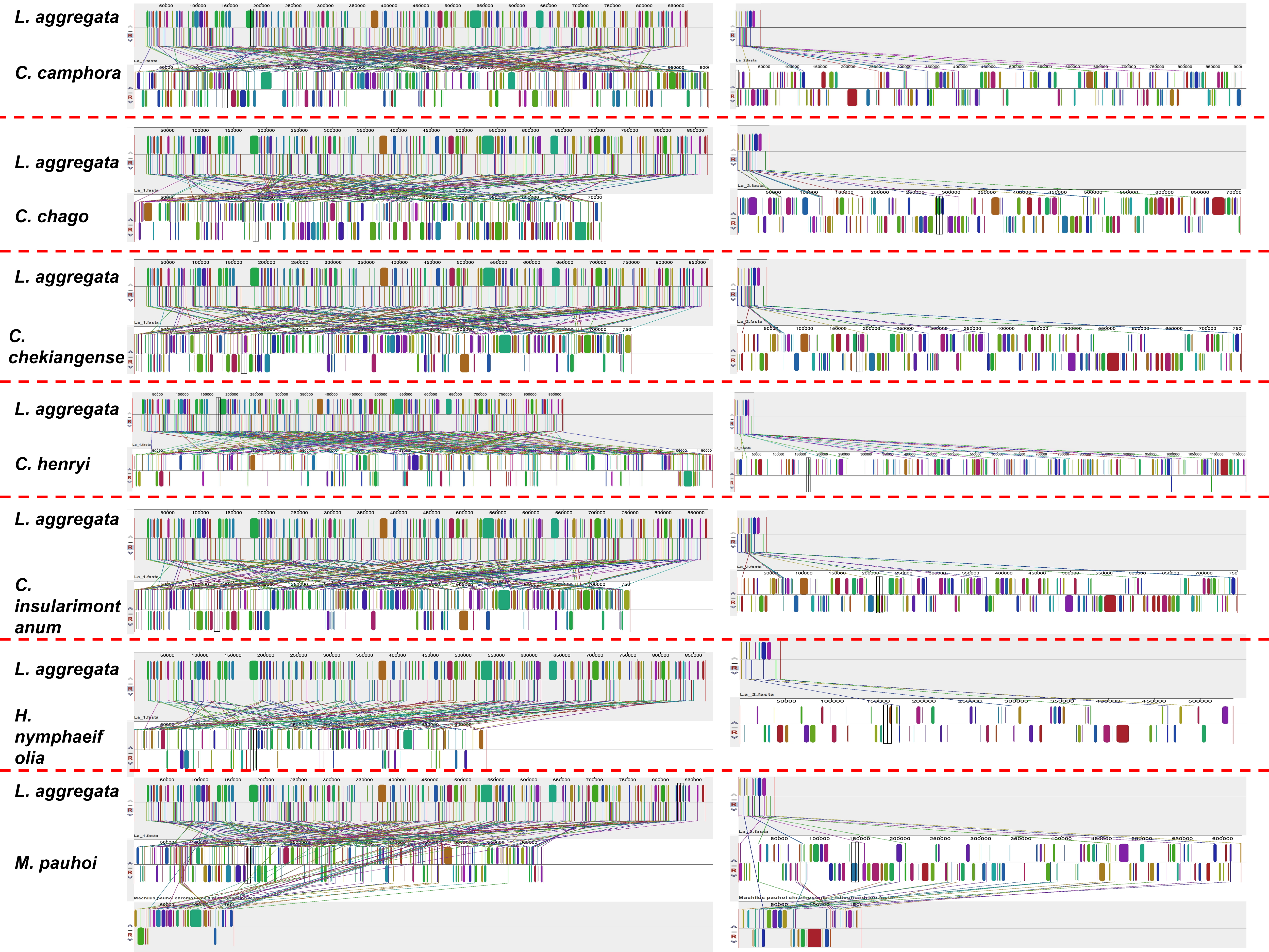


Figure S4. Colinearity analysis of 7 mitochondrial genomes of Laurales plants, with *Lindera aggregate* mitogenome as reference. Homogeneous fragments are represented by the same color, and fragments in the opposite order from the reference genome may have undergone rearrangement events.
